# Mobile Ground-Truth 3D Detection Environment for Agricultural Robot Field Testing

**DOI:** 10.3390/s25134103

**Published:** 2025-06-30

**Authors:** Daniel Barrelmeyer, Stefan Stiene, Jannik Jose, Mario Porrmann

**Affiliations:** 1 Faculty of Engineering and Computer Science, University of Applied Sciences Osnabrück, 49076 Osnabrück, Germany; s.stiene@hs-osnabrueck.de (S.S.); jannik.jose@hs-osnabrueck.de (J.J.); 2 Institute of Computer Science, Osnabrück University, 49090 Osnabrück, Germany; mario.porrmann@uni-osnabrueck.de

**Keywords:** agricultural robotics, ground truth, 3D-LiDAR, GNSS time synchronisation, multi-sensor fusion, autonomous field testing, geofencing

## Abstract

Safety and performance validation of autonomous agricultural robots is critically dependent on realistic, mobile test environments that provide high-fidelity ground truth. Existing infrastructures focus on either component-level sensor evaluation in fixed setups or system-level black-box testing under constrained conditions, lacking true mobility, multi-object capability and tracking or detecting objects in multiple Degrees Of Freedom (DOFs) in unstructured fields. In this paper, we present a sensor station network designed to overcome these limitations. Our mobile testbed consists of self-powered stations, each equipped with a high-resolution 3D-Light Detection And Ranging (LiDAR) sensor, dual-antenna Global Navigation Satellite System (GNSS) receivers and on-board edge computers. By synchronising over GNSS time and calibrating rigid LiDAR-to-LiDAR transformations, we fuse point clouds from multiple stations into a coherent geometric representation of a real agricultural environment, which we sample at up to 20 Hz. We demonstrate the performance of the system in field experiments with an autonomous robot traversing a 26,000 m^2^ area at up to 20 km/h. Our results show continuous and consistent detections of the robot even at the field boundaries. This work will enable a comprehensive evaluation of geofencing and environmental perception capabilities, paving the way for safety and performance benchmarking of agricultural robot systems.

## 1. Introduction

A lot of work is currently being carried out on autonomous systems, especially autonomous agricultural robots. From a process and safety point of view, there is a great need to test these developments thoroughly. The lack of test and benchmark environments with varying degrees of granularity hinders the independent evaluation of technology safety and performance. The creation of standards and norms, as well as certification, which simplify the development process and provide security, are heavily dependent on test environments and measurement regulations being developed and accepted [1].

This paper aims to evaluate and develop an approach to designing a test and benchmarking environment for evaluating the safety and performance of highly automated agricultural mobile machinery. Given the existence of methodologies and physical test infrastructures that enable the assessment of aspects such as environmental perception and the evaluation of detection capabilities at the sensor level [2], as well as the assessment of sensor concepts involving techniques such as sensor fusion and the integration of multiple sensors and algorithms [3], this paper focuses on the examination of system behaviour.

Similarly, at the system and application level, it is necessary to evaluate the performance or safety of critical functions by treating the entire system as a black box and testing it. Furthermore, at the application level, the system must be tested while performing a real task in a realistic work environment. Among the testing of safe system behaviour, geofencing and environmental perception stand out as particularly critical. A geofence is a polygon defined by geo-points that is analogous to a physical fence. In most cases, it defines the operational area for machinery. It is of great importance to guarantee that the machinery remains within the geofence. The localisation of agricultural machinery is highly dependent on GNSS, which can be influenced or manipulated by factors such as the surrounding environment. Therefore, it is essential to ensure that the machine remains within the designated working area or geofence at all times. This involves the creation of a test environment that can be employed to ascertain whether a machine remains within the geofence, despite the presence of various GNSS error sources. In terms of environmental perception, it is of critical importance to be able to perceive obstacles that were not known prior to the event and to avoid collisions.

This can be achieved by stopping the machine and bringing it into a safe mode or by triggering a re-planning, which allows the vehicle to bypass the object. The appropriate course of action may depend on the type of object. This would require not only the detection of the object but also its classification.

In general, for the purposes of testing and benchmarking, it is necessary to obtain information that is considered to be the ground truth, against which the information obtained by the agricultural mobile machine can be compared. Given the considerable diversity and variability in environmental conditions for agricultural robots (e.g., vegetation, field size, field environment), and given our objective of testing and benchmarking agricultural mobile robots at the system and application level, it is indispensable that the test environment we require be mobile in order to be deployed in the different environments.

This paper is structured as follows: Section 2 provides an overview of existing test environments in the area of environmental perception and geofencing, along with the associated test procedures. In Section 3, our custom-built test environment is described in detail, covering both the conceptual framework and the technical implementation. Section 4 focuses on the execution of a specific test, with a comprehensive presentation of results and observations. Finally, Section 5 discusses the positioning of our test environment in comparison to existing approaches, addresses identified system limitations, and outlines potential improvements for future development.

## 2. Related Work

The aim of this chapter is to provide an overview of existing test infrastructures and test methods. The overview focuses on infrastructures for outdoor applications, especially those that would be applicable to agricultural robots. It shows which functionalities and system granularities are addressed and where the limitations of existing systems lie. In particular, it will be examined to what extent the infrastructures, metrics and methods can be used to test functionalities such as geofencing and environmental perception of agricultural robots.

In any engineering discipline—whether dealing with software, electronics, mechanics, or an integration of these fields—a fundamental principle is the rigorous testing of complex systems. Complex systems are composed of multiple interdependent subsystems, and failures can emerge at various levels [4]. A malfunction may occur in an individual component (unit failure), during the integration of modules (integration failure), or even at the higher level when numerous subsystems interact in unforeseen ways (system-level or emergent failure) [5]. Moreover, when end users operate a system outside of its intended parameters, additional failure modes may be exposed.

This multi-layered nature of potential failures underscores the necessity for a comprehensive, multi-tiered testing strategy that spans from component-level tests through integration and system-level validation to end-user acceptance testing. Such an approach is critical not only for ensuring reliability but also for maintaining overall system safety and performance. Safety standards like IEC 61508 [6] and ISO 26262 [7] further emphasise the importance of this hierarchical testing paradigm in mitigating risks associated with complex, interconnected systems.

Komesker et al. [8] propose to adapt this testing paradigm for safety-related functions in autonomous working machines, such as agricultural robots. They suggest a V-model approach to validate the environment perception systems of these machines. They recommend to provide validation methods and test infrastructures for the different granularities, such as components (sensors), subsystems (sensor fusion), systems (sensor network) and applications (machine). Overall, the need for validation including test methods, metrics and infrastructures at different levels is emphasised. This is especially true for safety-related functions such as environmental perception and geofencing for autonomous working machines in outdoor environments such as agricultural robots.

Meltebrink et al. have developed a testbed [9] and a method [10] for evaluating sensor systems for the environmental perception of autonomous machines (in particular agricultural robots) under variable environmental conditions. The testbed allows a humanoid test subject to be moved on one portal and a carriage with different sensors to be moved on another portal in the plane, so that different relative movements occur. The aim is to demonstrate the ability of the different sensors to detect the test subject and thus to validate the suitability of single or multiple sensors in combination for environmental perception under different boundary conditions (relative motion, weather, sensor configuration, etc.). The testbed is site-specific and limited in size, which results in limitations. It can only be tested under the boundary conditions (e.g., weather conditions) that occur at the installation site. The test setup is specifically designed for sensor-level testing and therefore does not allow for system- or application-level testing.

Vaidis et al. [11] proposed a system which relies on a combination of three total stations and three prisms attached to a robot in an outdoor environment to provide accurate ground-truth (6 DOFs) pose information for the robot. A total station is a precision surveying instrument that combines an electronic theodolite for measuring horizontal and vertical angles with an electronic rangefinder for measuring distances and then calculating and recording the 3D coordinates of a target point. Under dynamic conditions, i.e., with a moving robot, an average position error of 10 mm and 0.6 deg can be achieved. The system is limited to a single object that can be tracked at the same time, which precludes the evaluation of the robot’s perception of the environment, such as object detection. Furthermore, the robot cannot be tracked straight out of the box. Three prisms have to be mounted in a specific way and calibrated to achieve the desired accuracy. The prisms also need to be in the line of sight of total stations. Consequently, a robot can only move within a certain range in an uneven outdoor environment in order for the pose to be tracked. The system does not allow the robot to be treated as a black box. The total stations are synchronised to the robots embedded computer clock via a long-range radio communication. Additionally the mounting of three prisms such that they are most likely visible for the total stations may disturb the sensors which are used for navigation and perception.

Krause et al. [12] proposed a testbed to collect data for object detection and collision avoidance in the context of environmental perception. The testbed consists of a rail-based carrier system equipped with a variety of environmental perception sensors. The carrier system moves through different sections of a real agricultural field with test targets placed in the different sections. The test targets are placed in fixed positions on magnetic anchors to ensure that the positions of the test objects remain the same for different test measurements. Ground-truth information for evaluation is provided by a dual Real-Time Kinematic (RTK) system on the carrier system. The target positions are surveyed with an RTK survey pole. Like the testbed of Meltebrink et al. [9], this setup also has the disadvantage of being stationary and therefore only covers a limited variance of possible agricultural boundary conditions. The variance is mainly due to the fact that measurements of the same scene can be taken throughout the vegetation period and over the seasons, and therefore under different weather conditions. It is important to note that such a testbed is no substitute for trials and tests with a real machine and an appropriate sensor setup. For example, the visual covers of the tractor and the implement cannot be adequately simulated by the testbed. In addition, there are boundary conditions in the real test that are difficult or impossible to reproduce on the testbed. Examples include the movement of the machine due to uneven ground, machine vibration, dust stirred up by the work process or other machines, and the variability of different fields and crops.

The project agROBOfood (2019–2024) [13], which was funded by the European Commission, had the objective of developing experiments for robots in the agrifood domain. In addition to other experiments, the French National Institute for Agricultural Research (INRAE) developed protocols for assessing the safety of agricultural robots in accordance with international standards, particularly ISO 18497 [14]. These are designated as an Agricultural Robot Performance Assessment (ARPA). The ARPA protocols comprise three distinct sets of procedures: ARPA1 for obstacle avoidance, ARPA2 for perception in challenging conditions, and ARPA3 for reliable geofencing. The protocols were developed initially by identifying and prioritising all potential causes of malfunction of the robot safety functions through the use of a Failure Mode and Effect Analysis (FMEA). In accordance with the findings and in alignment with the fundamental tenets of ISO 18497, the protocols were established. In order to ensure independence from GNSS reception conditions, a laser tracker is employed for the purpose of locating the robot. The ARPA1, ARPA2 and ARPA3 protocols are included in the following standard: Agricultural machinery and tractors–Safety of partially automated, semi-autonomous and autonomous machinery—Part 4: Verification methods and validation principles (ISO 18497-4:2024) [15]. Moreover, they are listed as exemplary test procedures for obstacle protection systems (ARPA1), for obstacle protection systems in rain and fog (ARPA2) and for an autonomous operating area (ARPA3). As with the setups described above, flexibility is limited. The system behaviour can only be evaluated based on the position (3 DOFs) of the mounted prism. No statements can be made about the orientation and geometry of the machine. In addition, only one object can be detected or tracked at a time.

Other system-level evaluation methods have been developed by Norris et al. [1]. They propose a system-level evaluation plan for field robots, covering different modes from manual to fully autonomous, test course layouts and protocols for evaluating safety, communication and behavioural performance in unstructured environments. However, their paper focuses on the test procedure and metrics for evaluation and does not provide a concept or technical implementation for an operational testbed or test environment.

Meltebrink et al. [16] have proposed a further testing and validation concept for agricultural robots. They present a concept where one or more Robot Operating System (ROS)-based validation stations or nodes are used. There should be a validation master to coordinate the validation process, and any number of additional stations for the actual validation process or several separate processes must be registered with the validation master. In a specific example, the collision avoidance of an agricultural robot is to be validated. Defined obstacles cross the robot’s path to the target position at different but fixed angles. The aim is to check whether the robot avoids the obstacles with a sufficient safety distance and reaches its target position. The verification will be based on information from two 2D-LiDAR validation stations that will monitor the test sequence. The specific technical implementation and evaluation software are not described in this article. The advantages of the presented concept lie in the modularity of the validation stations. However, it is not clear how; for example, the synchronisation of the data or the data exchange between the stations takes place. In addition, the use of 2D-LiDAR sensors means that only limited features are included in the point cloud and therefore less information can be obtained about the environment and the objects to be detected. This limits flexibility for other test scenarios. Aspects such as power supply, geo-referencing and communication are not discussed.

The review of existing test infrastructures highlights a clear progression from component-level sensor evaluations to more integrated, system-level methods. Sensor-focused testbeds, such as those of Meltebrink et al. [9,10], provide valuable insights into detection capabilities under controlled conditions but remain stationary and limited in spatial extent, weather variability and task realism. Survey instrument approaches, like described in Vaidis et al. [11] and laser tracker-based ARPA protocols [13,14], achieve sub-centimetre pose accuracy for a single target; however, they require extensive setup, fixed infrastructure, and are restricted to one object at a time. Rail-mounted carriers and dual-RTK systems (Krause et al. [12]) expand field realism but still lack true mobility and the ability to treat the robot as a black box.

Across the described approaches, three gaps emerge:

1. **Mobility and Flexibility:** No current solution combines the freedom to deploy in arbitrary fields with full self-sufficiency (power, communications, geo-referencing).
2. **Comprehensive System-Level Evaluation:** Many existing methods focus on sensor subsystems or impose artificial constraints, preventing holistic, black-box testing of autonomy functions such as geofencing and obstacle avoidance.
3. **Multi-Object Tracking:** While some setups achieve high positional accuracy, the ability to simultaneously track multiple objects’ full pose and extent (position, orientation, size) across a realistic and flexible agricultural process is not available.

These limitations motivate the need for a mobile, self-contained benchmarking environment capable of high-frequency, multi-sensor fusion and 9-DOF tracking of multiple targets in large, unstructured agricultural fields. In Section 3, we present our custom-built system designed to address these specific gaps.

## 3. System Overview

### 3.1. General Requirements of the Benchmark Field

This chapter aims to elaborate the general requirements for a test and benchmark field to evaluate the navigation and environmental perception performance and safety of highly automated mobile agricultural machinery given the limitations of existing test infrastructures, which were pointed out in the previous chapter. As mentioned in the introduction, the test and benchmark setup should be mobile. This means that it should be possible to use it in any agricultural field. The environmental conditions in agriculture are so diverse and subject to strong temporal and, above all, local changes that it is not possible to cover this diversity with a fixed, stationary infrastructure and, for example, to cover a few infrequent but possibly critical test cases (e.g., satellite blocked by trees and therefore no or poor GNSS signal close to the field boundary, combined with other events such as people moving close to the machine) that require this local flexibility. It is an inevitable consequence of this requirement that any physical infrastructure (e.g., sensors and other hardware) needed to perform the evaluations must also be mobile. From this follows the requirement that the system should be as self-sufficient as possible. Agricultural areas usually do not have fixed infrastructure for power supply and communication. This means that the mobile hardware must be able to supply itself with energy. It should also be noted that physical installations (such as laying cables) are required for communication.

Furthermore, as mentioned above, the focus should be on evaluating the system behaviour. This means that rather than testing individual modules or combinations of modules, the whole system should be tested in realistic scenarios or applications that allow evaluation of the intended functions. For this purpose, it should be possible to treat the system as a black box. This means that it is neither necessary nor desirable to communicate with the mobile agricultural machine during the test. The functions to be evaluated include geofencing and environmental perception. This results in several requirements, especially in terms of the data to be collected from the sensors. Firstly, it must be possible to determine the position of the mobile agricultural machine and track it over time. If only a specific reference point is tracked as the position, there is no guarantee that other areas of the sometimes large agricultural machine are not already outside the boundary, even though the reference point is still inside the boundary. This means that the data must also make it possible to detect and track the orientation of the object. In addition, there is no a priori knowledge of the dimensions of the object, and many of the highly automated agricultural robots being developed today consist of a combination of a tractor and implements. These attachments can change their configuration during the process. An example of this is the lifting of implements at the headland. It is therefore necessary to dynamically detect the dimensions of the agricultural machine or the combination of a tractor and implements. If the dimensions are understood as a scalar extension in three spatial axes (3 DOFs), in combination with position (3 DOFs) and orientation (3 DOFs), then the objective regarding the collected data can be summarised as the possibility of temporal detection of a single object or multiple objects in 9 DOFs.

Most agricultural machines and robots rely on GNSS for positioning and navigation [17,18]. Therefore, the working area (geofence) is also defined in a geodetic reference system (e.g., WGS84). In order to make the geofence data comparable to the position and orientation data described so far, it is necessary that the detection data can also be geo-referenced. For environmental perception, it is important that the robot can detect a priori unknown objects in its environment. This information can then be used to avoid collisions by adapting the behaviour of the machine if necessary (e.g., avoiding, reducing speed or stopping). In order to take action depending on the type of object, it is also necessary to be able to classify the object. In order to evaluate this functionality, it must be possible to detect and track multiple objects, so that a statement can be made about how many objects are in the robot’s environment, at what time and where. This should be possible on an area that allows real agricultural processes to be carried out. According to [19], almost two-thirds of the EU’s farms were less than 5 hectares (ha) in size. With this order of magnitude in mind, we define a minimum field size of 1 ha (equivalent to 10,000 square metres) to be monitored. This is necessary so that modern and larger machines and robots can also be used and detected for short periods of time.

Measurements should be made at a frequency that provides sufficient reference points at typical working speeds in agricultural operations. With regard to [20], which lists typical working speeds for agricultural machinery, the maximum speed considered is 15 km/h. This means that the typical speeds of operations such as untilage, seeding, fertiliser and pesticide application, and harvesting are covered with a sufficient buffer. The minimum requirement is a detection frequency of 5 Hz, which, at 15 km/h, would result in a distance between two successive measurement points of less than 1 metre.

In summary, the intended test and benchmark field should be mobile, self-sufficient and capable of tracking multiple objects in 9 DOFs (position, orientation and extension) over time with sufficient frequency and over an area large enough to carry out real agricultural processes. To clarify, ’capable’ in this context means that the selected hardware, particularly the sensors, must provide data that allows the described information to be obtained. It is explicitly not the aim of this work to provide the complete software backend for multi-object tracking. On the software side, the feasibility will be demonstrated by first performing single-object detection with data from real experiments. In other words, the detection of a single object in 9 DOFs will be carried out in a real agricultural context in successive measurements.

### 3.2. General Hardware Setup

In this chapter, we introduce a setup that is able to meet the requirements. Similar to the concept of Meltebrink et al. [16], we propose a system consisting of several identical mobile sensor stations, which can be flexibly distributed on the test field and remain there for the duration of the tests. Each sensor station has its own power supply in the form of a battery. Each station is equipped with high-resolution, long-range 3D-LiDAR to capture the geometry of the environment. The data obtained by the LiDAR sensors is used to track various objects. In addition, each station has a GNSS receiver with two antennas to measure the position and orientation of the stations in the geodetic reference coordinate system, thus providing geo-referencing. The GNSS receiver is also used to synchronise the sensor stations with each other by providing access to the precise time information from the satellites. In addition, each sensor station is equipped with an edge computer, which allows the sensor data to be pre-processed and stored locally. Due to the expected amount of data, the measurement data should be stored locally and then analysed offline. If a semantic understanding of the scene is required, for which the information provided by the LiDAR sensors is not sufficient, the sensor stations can be supplemented with cameras. However, we have not considered camera data in this work and have not included it in the hardware setup yet.

### 3.3. Calculated System Boundaries

**Figure 1:**
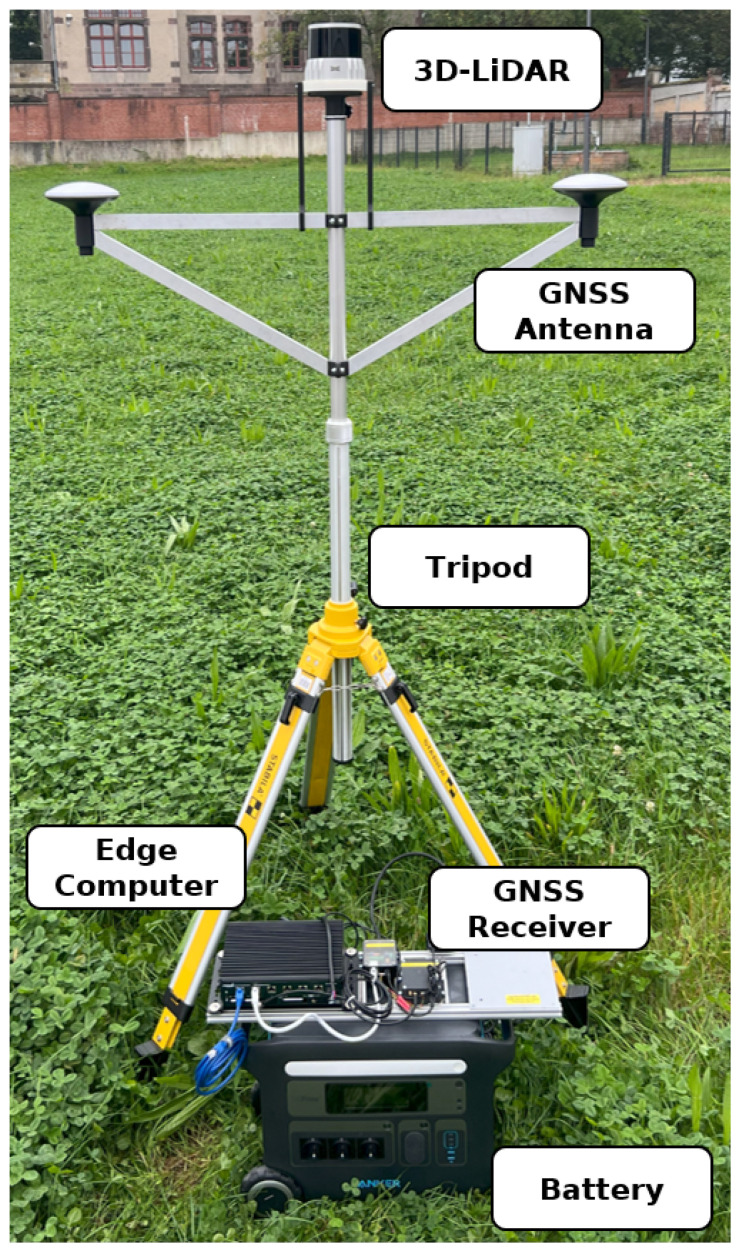
Picture of one assembled sensor station.

This chapter suggests specific hardware components for the previously presented setup and calculates the expected system limitations of the system. The chosen hardware is listed in Table 1. An image of the assembled sensor station is displayed in Figure 1.

**Figure 2:**
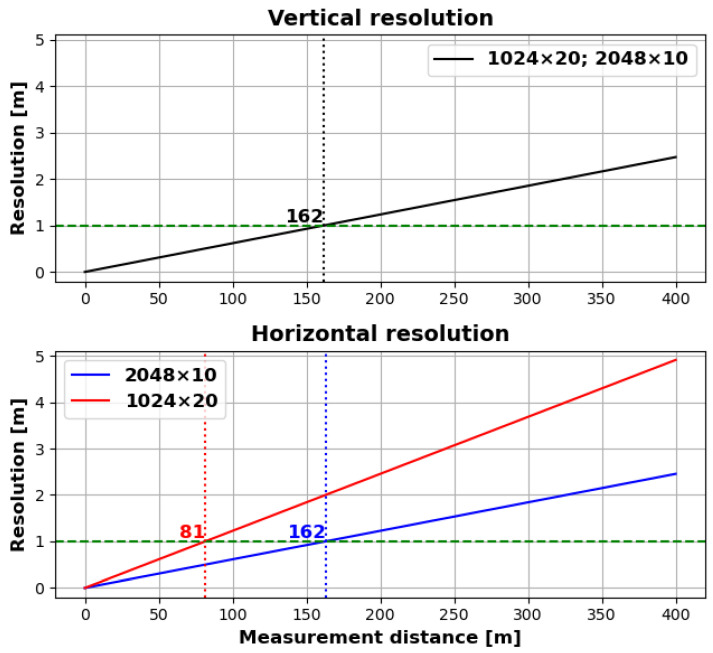
Increase in distance between measurement points (i.e., decrease in spatial resolution) in vertical and horizontal directions over nominal LiDAR measurement distance. The dotted lines indicate the distance at which a resolution of 1 metre is achieved.

The 3D-LiDAR is an Ouster OS2-128 with the following specifications. The vertical opening angle (field of view) is 22.5° with 128 layers and up to 2048 samples per layer. In each layer, points are recorded over the entire 360°. This consequently results in a decline in spatial resolution over the maximum nominal measurement distance of 400 m, as illustrated in Figure 2. The resolution of 2048 samples per layer enables the recording of measurements at a frequency of 10 Hz (2048 × 10 mode). It is possible to increase the frequency to 20 Hz. Nevertheless, only 1024 samples per layer are feasible in this instance (1024 × 20 mode). It is essential to ascertain the extent to which the frequency and resolution impact the ground-truth information. To evaluate the potential dimensions of the measurable areas, we set a limit of 1 metre for the minimum vertical and horizontal resolution per sensor station, assuming that an agricultural machine or robot can be detected in the combined point cloud of the sensor stations up to this resolution. This assumption needs to be verified. This assumed resolution results in a range of approximately 81m, which is limited by the horizontal resolution in the 1024 × 20 mode.

**Figure 3:**
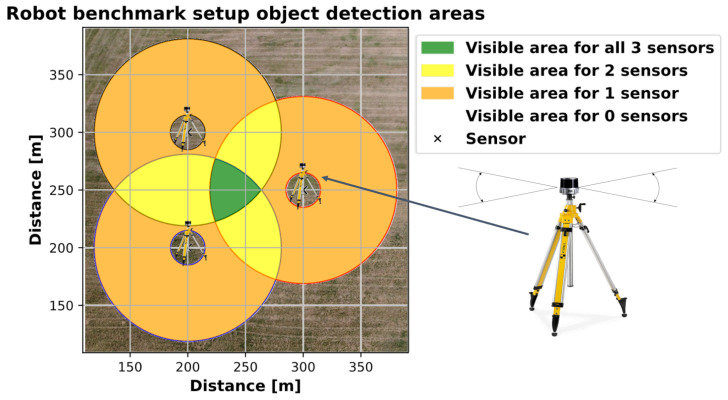
Visualisation of the potential object detection area, based on the assumed resolution threshold.

By integrating multiple high-resolution 3D-LiDAR sensors and positioning the sensor stations at diverse locations in the surrounding area, it is possible to obtain multiple reflections with high spatial resolution from different angles on the various objects in the scenery. We decided to set up three of the sensor stations. It is assumed that this is the minimum number of sensor stations required to provide an adequate spatial impression of the objects to be detected within a sufficiently large area for agricultural experiments. Additionally, this number provides a relatively cost-effective starting point for analysing the potential of sensor stations. Figure 3 illustrates the dimensions of the test environment, which are conducive to the evaluation of agricultural machinery and agricultural mobile robots. The monitored field encompasses an area of several 10,000 square metres (equivalent to 1 hectare or 2.47 acres).

The LiDAR position is adaptable in height, with the tripods’ mounting point able to be changed between 1.18 m and 3.0 m. This allows for the height to be adjusted in order to ensure that as many beams as possible can hit the objects in the area of interest. Furthermore, it allows for the potential to deal with uneven terrain with moderate elevations.

If the 3D-LiDAR sensors operate at 20 Hz and therefore deliver a new point cloud for each sensor every 50 ms, then it is expected that a new detection will also occur at the same frequency. However, the sensors do not deliver their measurements at the same absolute time, so three individual measurements to be merged will be spaced less than 50 ms apart, assuming no missing measurements and a stable measurement frequency. This can lead to distortions in the merged point cloud and affect the accuracy of the detection. It should be noted that a 50 ms offset represents a worst-case scenario. The algorithm used to assign time stamps at a stable frequency ensures that the time offset generally remains below 50 ms (see Section 3.4.2). The extent to which the unavoidable errors can be compensated for by merging the LiDAR data from the individual sensor stations needs to be examined. However, assuming a new object detection at every 50 ms, a reference point on the moving object at approximately 15 km/h could therefore move about 21 cm between two successive detections. We assume that using and fusing several sensor stations will sufficiently compensate for the error.

As one of the key requirements is for the sensor stations to be self-sufficient, it is essential that the batteries can supply the power required by the hardware for several hours in measurement mode. The batteries have a capacity of 2048 Wh. With a measured power consumption of about 53 W in measuring mode, operation for about 38 h is possible, allowing long test campaigns and leaving a large buffer for any additional consumers or more energy-intensive pre-processing steps on the edge computer. When measuring the total power consumption, the LiDAR was operated in the 1024 × 20 mode. According to the data sheet, 18–24 W are required for the LiDAR sensor in normal operation. The rest is mainly required for the operation of the edge computer.

### 3.4. Multi-Sensor Fusion

This chapter explains how to merge the data obtained from the three-dimensional LiDAR sensors and to detect a single object within the integrated point cloud. To achieve this, it is necessary to synchronise the 3D-LiDAR sensors in time and to calibrate the 3D rigid transformation between the different sensor frames, thereby enabling the presentation of the point clouds in a common frame. When deploying the sensor stations in a new environment, no area-specific configuration is necessary. The timestamped LiDAR and GNSS data are recorded in ROS 2 bags in the mcap file format by the edge computer on every sensor station and are subsequently processed offline with the data from the other sensor stations. Given the considerable volume of data generated by, for instance, the 3D-LiDAR sensors, online real-time exchange and computation on the raw data are not feasible.

Following the data acquisition, an offline analysis is conducted. Firstly, the calibration procedure is carried out. The goal is to obtain accurate transformations for the sensor nodes such that the point clouds are aligned and that it is possible to compute the correct GNSS position for each point. To register the point clouds, an Iterative Closest Point (ICP) alignment is performed as described in [21] to identify the rigid 3D transformations between the sensor frames. In order to find an accurate solution and to converge in an acceptable time, the ICP algorithms require an estimation of a rough transformation as input. In this case, the position and orientation information from the GNSS receivers is used to compute the rough transformation. Finally, a global correction of the transformations, which takes the GNSS positions of all sensor nodes into account, is performed to ensure an accurate GNSS reference for each point.

With these transformations, it is possible to align the individual point clouds of each sensor node, which are linked by the timestamp. In the merged point cloud, the points belonging to the robot are now extracted using a set of algorithms and filters, as described in Section 3.5. Finally, it is possible to calculate a 3D-orientated bounding box of the robot point cloud. The Open3D framework [22] was used to manipulate, analyse and display the point cloud data.

#### 3.4.1. Time Synchronisation

In order to achieve adequate sensor fusion, it is essential to ensure precise time synchronisation between the various sensor stations and the disparate sensors. In the proposed system, this is achieved through the utilisation of the time information provided by the GNSS receivers. The technique is commonly employed in autonomous systems, given that GNSS systems are known to provide accurate time information [23,24,25,26]. This is due to the fact that the satellites have highly accurate atomic clocks on-board, which are synchronised to Coordinated Universal Time (UTC) [27]. This is a standard that they need and have to send anyway, so that GNSS receivers can determine an exact position based on the transit time differences of the signals, among other things. The sensor stations are equipped with simpleRTK3B boards that incorporate a Septentrio Mosaic-H chip. These boards are capable of providing a digital Pulse Per Second (PPS) signal, which can be employed to synchronise other devices. The pulses occur precisely once per second within the UTC time frame. As the pulse itself does not contain absolute time information, this is obtained from GNSS data (National Marine Electronics Association (NMEA) GPRMC standard sentence). According to the datasheet, the mosaic-H module is able to propagate a PPS signal with a time precision of 5 ns.

The Ouster OS2-128 provides direct interfaces for the digital PPS signal (SYNC_PULSE_IN) and NMEA sentences (MULTIPURPOSE_IO, configurable as Universal Asynchronous Receiver/Transmitter (UART) interface) in the form of a JST connector, allowing the connection of an external GNSS receiver, thus enabling precise time stamping of sensor data independent of any network infrastructure. Furthermore, the LiDAR is capable of supplying the energy required for the GNSS receiver via the JST connector. The LiDAR can be configured to utilise a time synchronisation input signal to synchronise its clock and timestamp the obtained LiDAR data directly within the dispatched User Datagram Protocol (UDP) data (minimum timestamp increment is 10 ns).

The advantages of this synchronisation method are that it provides cost-effective access to the highly accurate atomic clocks of the satellites, thereby offering the potential to provide an accurate signal for time synchronisation. Furthermore, the necessity for establishing a local wireless network (due to the unfeasibility of a wired network) or an internet connection is negated. Therefore, as described in [23], this solution is independent of network outages, latency, or jitter, which are common issues with network-based protocols like Network Time Protocol (NTP) and Precision Time Protocol (PTP). It only depends on at least one satellite in sight, which should be almost always visible due to the intended area of application and the possibility of positioning the sensor stations free from obstructions.

#### 3.4.2. Point Cloud Registration and Geo-Reference

**Figure 4:**
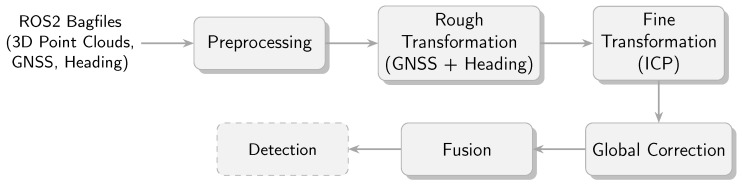
Schematic overview of the multi-sensor fusion pipeline.

Given the stationary nature of the sensor nodes during data acquisition, the point cloud registration needs to be conducted only once in order to obtain the required transformations. The process is displayed in Figure 4. Given the importance of an initial alignment for ICP registration, we compute preliminary transformations based on the GNSS position and heading data provided by the GNSS receivers on the sensor nodes. The GNSS positions are projected into a local Cartesian coordinate system using the Universal Transverse Mercator (UTM) projection methods from [28]. The sensor nodes are assigned numerical identifiers. The local origin is defined in accordance with the position of the initial sensor node. In order to ensure compatibility with the ROS standards, the system adheres to the East-North-Up (ENU) convention [29]. The resulting transformations represent the translation and rotation from the ENU coordinate system to each sensor node coordinate system, which is identical to the LiDAR coordinate system of the respective sensor node.

**Figure 5:**
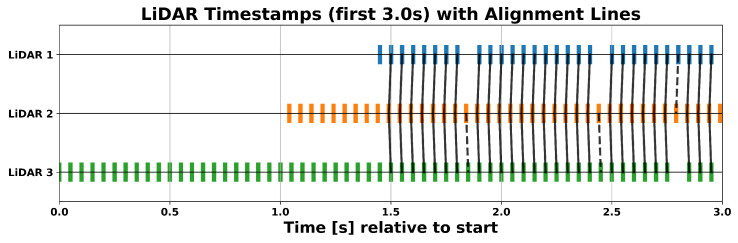
Alignment of LiDAR timestamps over the first 3 s of measurement using Dynamic Time Warping. Vertical lines connect the closest matching timestamps between each sensor’s stream and the reference (LiDAR 1), illustrating how DTW compensates for slight timing offsets and variable sampling intervals before ICP registration.

Prior to the point cloud registration, it is necessary to search for related point clouds using the time stamps. This is achieved through the utilisation of a Dynamic Time Warping (DTW) algorithm [30] in conjunction with constraints, including a maximum time tolerance between two corresponding time stamps and the requirement that each time stamp is only employed once. The result of the time stamp alignment for the first 3 s of a measurement is shown in Figure 5.

**Figure 6:**
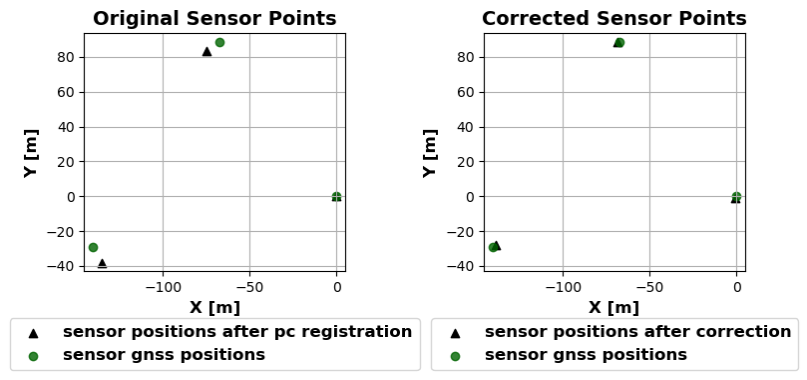
Comparison of sensor positions before (**left**) and after (**right**) the final alignment correction. Black triangles show sensor positions derived from the point cloud registration, while green circles represent the corresponding GNSS-measured positions. After applying the alignment corrections, the sensor positions align more closely with their GNSS references.

**Figure 7:**
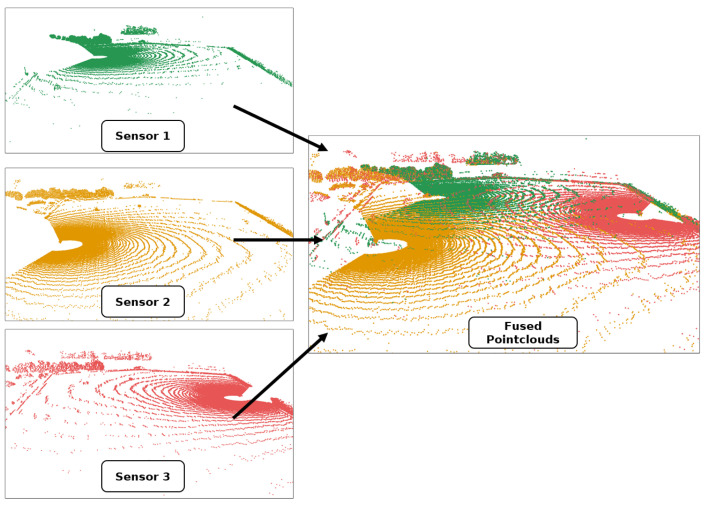
Individual point clouds captured by three sensors (green, orange, and red), and their combined (fused) point cloud, which is subsequently used for the detection.

Subsequently, a point-to-plane ICP algorithm [21] is employed to achieve precise alignment between the point clouds. The point clouds of the second and third sensor nodes are aligned with the point cloud of the first sensor node in the ENU frame. The transformation describing the position and orientation of the first point cloud is derived from the preliminary orientation based on the GNSS heading information. As the transformation depends only on the position and heading data obtained by the GNSS receiver of the first sensor node, small inaccuracies, particularly in the heading measurement, can result in a rotational misalignment in the quality of the geo-reference when a position is projected back to the World Geodetic System 1984 (WGS84) coordinate system. To illustrate, a rotation error at the first sensor node would result in progressively larger discrepancies between the measured GNSS positions when the coordinates are transformed to a local Cartesian system. This occurs despite the ICP performing well and resulting in an accurate alignment. Up to this point, the GNSS positions of the other sensor nodes, known with good accuracy, were only used to provide an approximate initial transformation for the point cloud registration. In the final step, an attempt is made to match the sensor positions with the GNSS positions of the sensors as closely as possible after registering the point cloud. As displayed in Figure 6, the transformations are corrected with the result of the alignment. Qualitative comparisons with recorded robot GNSS positions have shown that geo-referencing errors can be compensated for by taking into account GNSS data from all the sensor stations. The final result of the registration and fusion into a common point cloud is shown in Figure 7 for three individual point clouds that belong together in time.

### 3.5. Object Detection

With the final transformations computed, the next step is to perform the object detection. The time-stamped point clouds from each sensor node are first fused into a single point cloud as described. This fusion is carried out twice: once for the current scan and once for a reference scan. The reference point cloud is recorded separately during a measurement in an environment free of dynamic objects, ensuring that only static elements are present.

In addition to these two point clouds (fused reference point cloud and fused point cloud at the current time stamp), the detection algorithm receives several other inputs: an area of interest (e.g., the borders of an agricultural field), the sensor node positions and, if available, a previously detected bounding box. The primary goal is to isolate the points corresponding to the object of interest (for example, an agricultural robot) and to compute a 3D-oriented bounding box around these points. To achieve this, a series of rules and filters are applied to discard irrelevant data. Suitable methods are generally available in the Open3D framework [22]. The algorithm is consciously designed without any machine learning approaches to keep the results as explainable as possible.

Initially, all points located outside the area of interest are removed. LiDAR measurements—such as those caused by operator movements within a predefined cubic region around each LiDAR sensor—are also filtered out. The subsequent procedure depends on whether the object’s previous position is known:

1. **Unknown Prior Position:**
  If no previous position is available, the normals for the remaining points are calculated. The floor is then filtered out based on the direction of these normal vectors. For each remaining point, a corresponding point in the reference cloud is identified. If the distance between them exceeds a predefined threshold, it is assumed that the point belongs to a new object that was absent during the reference scan. Afterward, statistical outlier removal is applied, followed by clustering to isolate the points associated with the robot.
2. **Known Prior Position:**
  When a prior robot position is known, a local area around this position is extracted. Within this local area, an equation representing the ground plane is computed, and reflections on the ground are filtered out using a threshold distance from the computed plane. Subsequent processing—statistical outlier removal and clustering—proceeds in the same manner as in the first case.

Differentiating between these two approaches has distinct advantages. The local area method allows for more accurate ground filtering, as a precise plane equation can only be computed when a previous position is available. Conversely, the normal-based approach has the advantage of a global search for objects, although it may inadvertently discard useful data (such as reflections on the target object).

**Figure 8:**
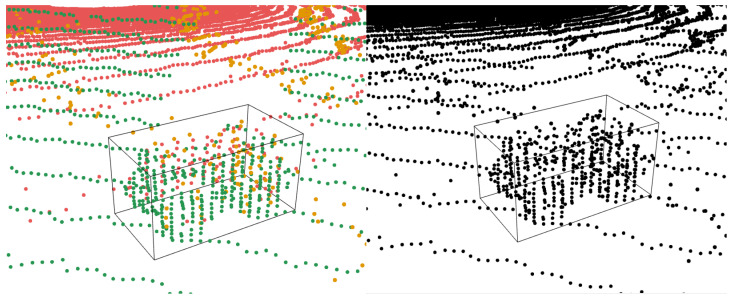
Visualisation of fused 3D point cloud data with an oriented bounding box highlighting the tracked robot. The left image shows colour-coded data points from multiple sensors, while the right image displays a monochrome version of the same data for clarity.

The final step is to compute a 3D-oriented bounding box for the clustered points. Figure 8 shows an exemplary result. If a robot with an attachment is to be detected, the robot and its attachment would be recognised as a single, connected object. This would increase the object’s dimensions. Consequently, more points would be found on the object, resulting in a larger cluster and ultimately a larger bounding box.

## 4. Experimental Results

**Figure 9:**
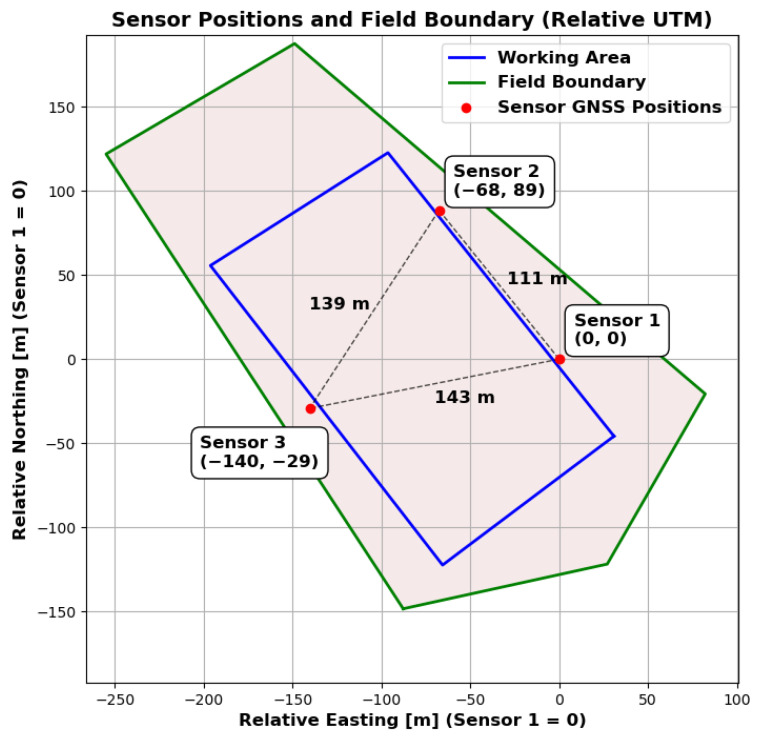
Schematic diagram of the experimental setup used for testing under real conditions. Sensor positions, field boundaries and working areas are shown in the local ENU coordinate system. The origin is at sensor 1. The x-axis is oriented to the east and the y-axis to the north.

**Figure 10:**
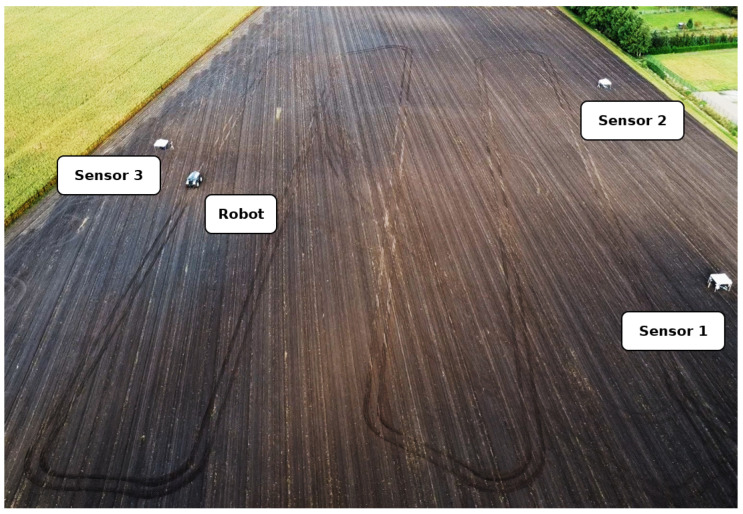
Drone image during the implementation of the described experiment on a real agricultural field. The positions of the sensors and the pavilions directly behind the sensors can be seen. The robot can also be seen following its planned path (tracks are visible on the field).

**Figure 11:**
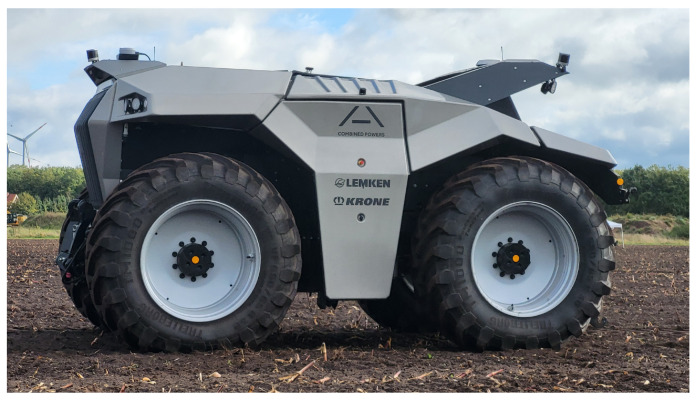
The autonomous agricultural robot used in the field experiment. The vehicle was provided and supervised by Maschinenfabrik Bernard Krone GmbH & Co. KG and developed in collaboration with LEMKEN GmbH & Co. KG as part of the Combined Powers project.

**Figure 12:**
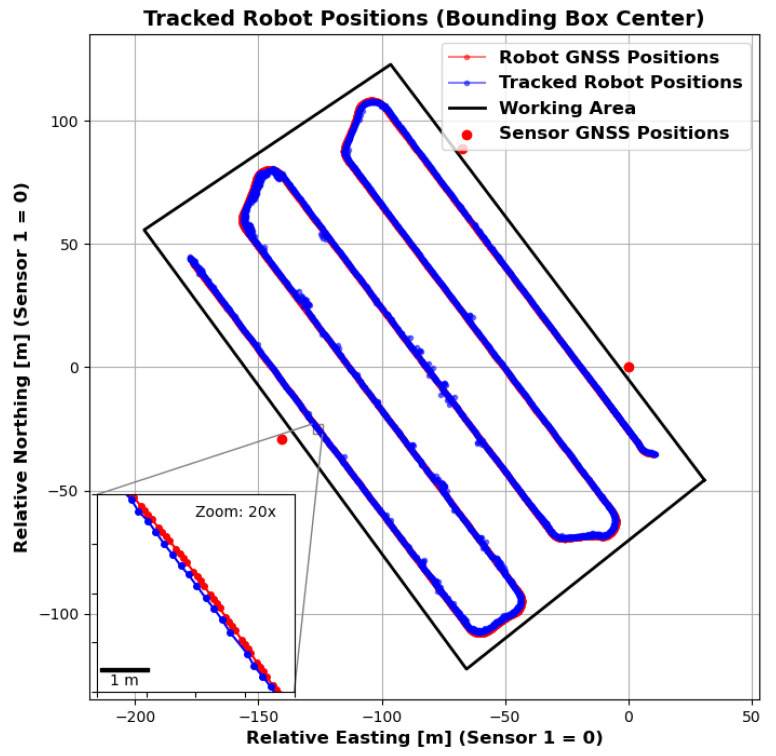
Detection results (centre of the bounding box) of a route travelled in the field together with the robot’s GNSS positions in one display. The enlarged section shows deviations between the position tracked by the sensor nodes and the position measured by the robot via GNSS.

An experiment was carried out to test the ability of the proposed system to track an agricultural robot on a real agricultural field. The experimental setup is shown in Figure 9 and Figure 10. A partial area (approximately 26,291 square metres) of a harvested field was selected for monitoring. Stubble cultivation already took place after the maize crop was harvested. The sensor stations were placed in a triangular arrangement outside the above-mentioned area and therefore outside the working area. The robot for the test was provided and supervised by the company Maschinenfabrik Bernard Krone GmbH & Co. KG (48480 Spelle, Germany), and it is shown in Figure 11. It is an autonomous agricultural robot developed in collaboration with LEMKEN GmbH & Co. KG (46519 Alpen, Germany) as part of the Combined Powers project [31]. The robot measures 5.5 m × 3.0 m × 2.9 m (length × width × height). It has a rear and front linkage for attaching implements. In the test, the robot was operated without any attachments. The route planning for the partial area was carried out by Krone in advance. The robot’s timestamped GNSS measurement data logs were provided. Figure 12 shows the detection results of one pass. The robot travelled at speeds of up to 20 km/h during this pass. The figure shows the centre of the detected bounding boxes using the proposed approach and the GNSS positions recorded by the robot. The enlarged area shows that the positions are already close to each other, even if they are not recorded or calculated with respect to a specific reference point (e.g., the position of the GNSS antenna mount, which does not correspond to the centre of the robot’s bounding box). However, it should also be noted that there are slight outliers along the detected route.

**Figure 13:**
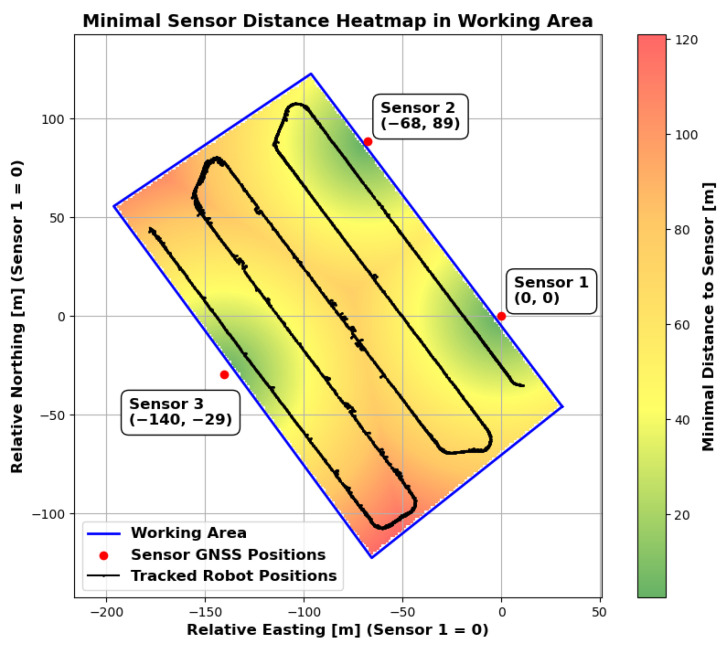
Minimal sensor distance heatmap in the working area. The polygon (blue) outlines the working area, and the colour gradient indicates the minimal distance to the nearest sensor (from green = shorter distance to red = longer distance). Red dots mark the sensor positions. The black trace shows tracked robot positions within the area.

**Figure 14:**
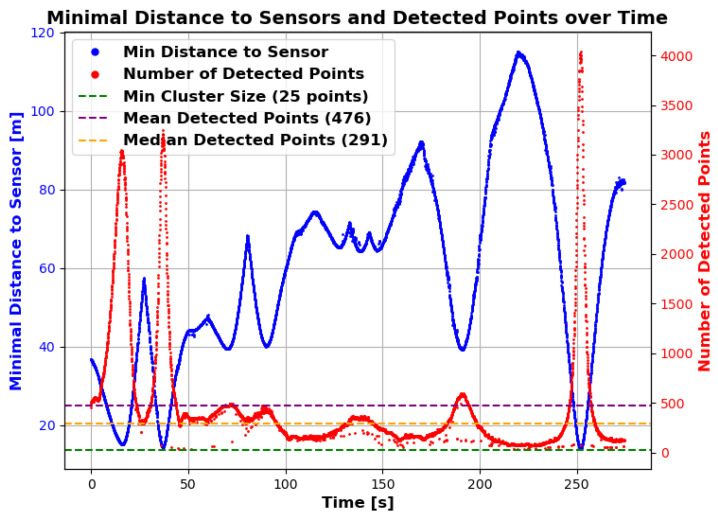
Minimal distance to the sensor and number of detected points over time. The blue curve (left y-axis) shows the minimal distance to the sensor, and the red curve (right y-axis) shows the number of detected points. The horizontal dashed green line indicates the minimum cluster size (25 points). The horizontal dashed orange line indicates the mean number of detected points (476 points), and the horizontal dashed purple line indicates the median number of detected points (291 points).

The sensors were deliberately placed at the distances shown. No distance between two sensors is greater than approximately 162 m, which, with the robot in the exact centre, still ensures a horizontal and vertical resolution of at least one metre for the LiDAR sensors. However, there are areas in the selected work area where the minimum distance to one of the sensors is greater. Figure 13 shows the distance to the nearest sensor for the whole working area as a heatmap. It can be seen that there are areas where the distance is greater (up to 120 m), particularly in the corners of the work area. Nevertheless, stable detection was achieved even in these areas. However, Figure 14 shows that the number of points on the robot (LiDAR measurements) drops to around 80 at these distances. In addition, the positioning of the robot in relation to the sensor stations located at the corners of the working area minimises the spatial impression of the object to be detected. The robot obscures parts of itself from all available sensor stations. These effects make stable detection in the peripheral areas more difficult, which is likely to result in reduced positional accuracy. However, it is not meaningful to make a quantitative comparison between the recorded GNSS path of the robot and the detected bounding box centre at this point. As previously mentioned, these two measurements do not refer to the same reference point. Furthermore, the system will be used in the future to evaluate whether the robot remains within its set working area. Therefore, validation should be carried out independently and not in relation to the robot’s positional data. Currently, this only allows for a qualitative comparison. To be protected against noise and measurement errors, a minimum number of points for clustering must be specified at the end of the object detection pipeline in order for a cluster to be detected as such. In the current implementation, a threshold of 25 is used, with which the robot could be detected stably over the entire field. Measured against the total number of points available, 80 points are already relatively close to this limit. This in turn suggests that the extent of the field is close to the maximum area that can be monitored. An alternative is to further reduce the minimum cluster size, but this would also increase the vulnerability to false detections. As expected, the number of points found is particularly high when the robot passes close to the sensor stations. The mean number of points is 476, but this is influenced by the peaks when passing the stations and is therefore only partially representative for the whole area. The median is 291 points and seems to be a better measure of the available points.

It has been shown that it is possible to detect an agricultural robot with the described size in a field with the chosen hardware setup and methods, e.g., for time synchronisation and sensor data fusion. Both the robot and the field have realistic and real-world dimensions, emphasising the relevance of the system. This will address the limitations of existing test methods and infrastructure, as outlined in Section 2. None of the described methods offer the possibility of detecting one or more objects in realistic-size flexible agricultural areas with a completely mobile, self-sufficient, 9-DOF test setup, thereby enabling system-level evaluation that treats objects, and particularly the robot, as black boxes. Therefore, the proposed system represents a valuable addition to existing test methods that focus on component- or sensor-level evaluations.

## 5. Discussion

This chapter aims to discuss the results of the experiments in the context of the requirements that have been set. The experiment described above demonstrated the basic suitability of the system developed and the hardware selected. With the three sensor stations set up, it is possible to generate measurement data that can be used to detect an object in nine degrees of freedom. This has been demonstrated in an agricultural context. It is possible to detect an agricultural robot in fields of a practical size. The system is highly scalable. It would be possible to add more stations of the same design to improve the detection quality and cover even larger areas. The sensor stations are mobile and self-sufficient. There is no dependence on a fixed infrastructure for communication or power supply. The methods for time synchronisation and, based on this, sensor data fusion are able to generate consistent point clouds based on the individual measurements of the sensor stations. It should be emphasised that even the parts of the point cloud that come from the dynamically moving object (up to 20 km/h) are consistent. There are no significant distortions that would make the robot detection much more difficult. This indicates that the measurement frequency, the quality of the time synchronisation, and the quality of the point cloud registration are of sufficient quality. This is initially a qualitative assessment. Further experiments and evaluations will be necessary to better quantify the quality. The experiments have had some limitations. So far, only a relatively large agricultural robot has been studied as an object. In the future, the system should be able to track several objects of different sizes at the same time. Relevant objects, such as other machines, people and animals, or comparable test objects, have significantly smaller dimensions than the agricultural robot under investigation. It needs to be investigated whether and to what extent the possible monitored area needs to be reduced in order to ensure stable tracking of smaller objects and multiple objects at the same time. In addition, only one robot without attachments has been studied in a field after harvest and after stubble cultivation. It is important to consider how, for example, fields with vegetation and the use of implements affect the detection result. In addition to quantifying the quality of the individual steps, the final result needs to be independently quantified and validated. In the experiments conducted so far, only the positioning result can be compared with the GNSS measurements provided by the agricultural robot. However, since the medium-term goal is to make an independent statement about the state or behaviour of this robot, it is necessary to work on an independent validation procedure. Both the quantification of the quality of the individual steps and the independent evaluation of the final result can be used to develop targeted improvement measures for accuracy and stability, e.g., against outliers. It should be noted that a mobile experimental environment was created that has the potential to enable statements to be made about the performance and safety of mobile agricultural robots at system and application levels. The developed system could make an important contribution to the development of important and critical functions for mobile, autonomously driving and working agricultural robots, such as environmental perception and geofencing.

## 6. Outlook

The previous chapter discussed the limitations of the experiments carried out so far and pointed out what should be improved. Based on this, future work should explore the tracking of multiple objects of different sizes, especially smaller ones. The problem of data association needs to be considered. What are the most suitable methods to satisfactorily solve the problem of associating the corresponding detected objects in successive frames in an agricultural context with the possible objects that may occur? This is particularly the case when multiple objects move close together or cross each other, obscuring the view of individual sensor states on objects. Further work should also be invested in investigating the quality of time synchronisation and point cloud registration. In addition, as mentioned above, the quality, i.e., the accuracy of the overall result, needs to be evaluated and validated independently of any robot data provided. For example, total stations with assured accuracies could be used for distance measurements. With these results, the system can be improved to achieve the highest possible accuracy when tracking multiple objects in the future. To this end, it should be possible to track a specific reference point in the robot’s point cloud. This could be achieved by providing or creating an existing 3D file (e.g., a mesh) which is then matched to the final clustered points to provide better information about the position of a specific reference point. In addition to that, since the measured reflections only partially capture the true shape of the object, determining an accurate bounding box in terms of position, orientation, and extent is nontrivial. If a digital representation of the robot or object is available, fitting this model to the observed points can provide a practical solution for deriving a more accurate bounding box. In principle, further experiments and tests need to be carried out that take into account parameters such as the number and size of objects, vegetation and the use of attachments, thereby covering a greater variance. Concerning the vegetation, further experiments should initially focus on arable farming and investigate factors such as the influence of crop height on detection quality. Other types of cultivation, such as orchards and vineyards, present a significant challenge due to the potential obstruction of sensors by vegetation. This may necessitate the incorporation of additional data sources, such as drone imagery. Subsequent studies could also investigate the integration of more sensors of the same or different modalities (such as cameras and radars) and their effect on tracking quality, robustness to environmental conditions, and semantic knowledge of the landscape and the objects within it.

## Figures and Tables

**Table 1:** Hardware components of a sensor station.

Name	Model	Manufacturer
3D-LiDAR	OS2-128	Ouster, Inc. (350 Treat Avenue, San Francisco, CA 94110, United States of America)
GNSS Receiver	simpleRTK3B	ArduSimple (Carrer Pau Casals 10, 3a planta AD500 Andorra la Vella, Andorra)
	(mosaic-H)	(Septentrio N.V.) (Greenhill Campus Interleuvenlaan 15i, 3001 Leuven, Belgium)
GNSS Antenna (2×)	AS-ANT2B-CAL-L1L2-15SMA	ArduSimple (Carrer Pau Casals 10, 3a planta AD500 Andorra la Vella, Andorra)
Battery	PowerHouse 767	Anker Innovations Technology Co., Ltd (Room 701, Bldg 7, Zhongdian Software Park, 39 Jianshan Road, Hi-tech Zone, Changsha City, Hunan Province, China)
Tripod	BST-K-L	STABILA Messgeräte Gustav Ullrich GmbH (Landauer Straße 45 76855 Annweiler, Germany)
Edge Computer	NRU-220S-JAO64G	Neousys Technology Inc. (11F., No. 198, Jian 8th Rd., Zhonghe Dist., New Taipei City 235042, Taiwan)
	(Jetson AGX Orin)	(NVIDIA) (2788 San Tomas Expressway Santa Clara, CA 95051, United States of America)

## Data Availability

The datasets presented in this article are not readily available due to time constraints and technical issues. This is because large amounts of data are generated in the form of rosbags with each measurement. However, it is generally possible to gain access to the data. Enquiries should be addressed to the corresponding author, Daniel Barrelmeyer.

## Abbreviations

The following abbreviations are used in this manuscript:

ARPA	Agricultural Robot Performance Assessment
DOFs	Degrees of Freedom
DTW	Dynamic Time Warping
ENU	East–North–Up
FMEA	Failure Mode and Effect Analysis
GNSS	Global Navigation Satellite System
ICP	Iterative Closest Point
LiDAR	Light Detection And Ranging
NMEA	National Marine Electronics Association
NTP	Network Time Protocol
PPS	Pulse Per Second
PTP	Precision Time Protocol
ROS 2	Robot Operating System 2

RTK	Real-Time Kinematic
UTM	Universal Transverse Mercator
UDP	User Datagram Protocol
UTC	Coordinated Universal Time
WGS84	World Geodetic System 1984

## References

[1] Norris W.R., Patterson A.E. (2019). System-Level Testing and Evaluation Plan for Field Robots: A Tutorial with Test Course Layouts. Robotics.

[2] Komesker M., Meltebrink C., Ebenhöch S., Zahner Y., Vlasic M., Stiene S. (2024). Validation Scores to Evaluate the Detection Capability of Sensor Systems Used for Autonomous Machines in Outdoor Environments. Electronics.

[3] Krause J.C., Martinez J., Gennet H., Urban M., Herbers J., Menke S., Röttgermann S., Hertzberg J., Ruckelshausen A. AI-TEST-FIELD—An Agricultural Test Environment for Semantic Environment Perception with Respect to Harsh And Changing Environmental Conditions. Proceedings of the 2023 ASABE Annual International Meeting.

[4] Leveson N.G. (2012). Engineering a Safer World.

[5] Hodel K.N., Da Reinaldo Silva J., Yoshioka L.R., Justo J.F., Santos M.M.D. (2022). FAT-AES: Systematic Methodology of Functional Testing for Automotive Embedded Software. IEEE Access.

[6] (2010). Functional Safety of Electrical/Electronic/Programmable Electronic Safety-Related Systems.

[7] (2018). Road Vehicles—Functional Safety.

[8] Komesker M., Meltebrink C., Ebenhöch S., Stiene S. V-Model Approach for Developing Safe Environment Perception Systems for Autonomous Machinery. Proceedings of the LAND.TECHNIK AgEng 2024.

[9] Meltebrink C., Ströer T., Wegmann B., Weltzien C., Ruckelshausen A. (2021). Concept and Realization of a Novel Test Method Using a Dynamic Test Stand for Detecting Persons by Sensor Systems on Autonomous Agricultural Robotics. Sensors.

[10] Meltebrink C., Komesker M., Kelsch C., König D., Jenz M., Strotdresch M., Wegmann B., Weltzien C., Ruckelshausen A. (2022). REDA: A New Methodology to Validate Sensor Systems for Person Detection under Variable Environmental Conditions. Sensors.

[11] Vaidis M., Giguere P., Pomerleau F., Kubelka V. Accurate outdoor ground truth based on total stations. Proceedings of the 2021 18th Conference on Robots and Vision (CRV).

[12] Krause J.C., Iqbal N., Niemeyer M., Thy B., Plagge L., Hollmeier H., Ruckelshausen A., Röttgermann S., Tauber A., Herbers J. AI-TEST-FIELD—A Test Environment for the Automated Evaluation of Methods for Robust and Reliable Environment Perception. Proceedings of the LAND.TECHNIK AgEng 2023.

[13] AgROBOfood Project|AgROBOfood. https://agrobofood.eu/about/project.

[14] (2018). Agricultural Machinery and Tractors—Safety of Highly Automated Agricultural Machines—Principles for Design.

[15] (2024). Agricultural Machinery and Tractors—Safety of Partially Automated, Semi-Autonomous and Autonomous Machinery—Verification Methods and Validation Principles.

[16] Meltebrink C., Linz A., Ruckelshausen A. ROS-basiertes Validierungskonzept für Sicherheitskonzepte von autonomen Agrarrobotern. Proceedings of the 36. GIL-Jahrestagung.

[17] Bechar A., Vigneault C. (2016). Agricultural robots for field operations: Concepts and components. Biosyst. Eng..

[18] Yao Z., Zhao C., Zhang T. (2024). Agricultural machinery automatic navigation technology. iScience.

[19] Eurostat Farm Indicators by Legal Status of the Holding, Utilised Agricultural Area, Type and Economic Size of the Farm and NUTS 2 Region. https://ec.europa.eu/eurostat/statistics-explained/index.php?title=Farms_and_farmland_in_the_European_Union_-_statistics.

[20] (2011). Agricultural Machinery Management Data.

[21] Chen Y., Medioni G. (1992). Object modelling by registration of multiple range images. Image Vis. Comput..

[22] Zhou Q.Y., Park J., Koltun V. (2018). Open3D: A Modern Library for 3D Data Processing. arXiv.

[23] Hasan K.F., Feng Y., Tian Y.C. (2018). GNSS Time Synchronization in Vehicular Ad-Hoc Networks: Benefits and Feasibility. IEEE Trans. Intell. Transp. Syst..

[24] Hasan K.F., Feng Y., Tian Y.C. (2023). Precise GNSS Time Synchronization with Experimental Validation in Vehicular Networks. IEEE Trans. Netw. Serv. Manag..

[25] Müller P., Berger D., Sarperi L. (2025). Nanosecond Time Synchronization over a 2.4 GHz Long-Range Wireless Link. Sensors.

[26] Albrektsen S.M., Johansen T.A. (2018). User-Configurable Timing and Navigation for UAVs. Sensors.

[27] Kaplan E.D., Hegarty C. (2017). Understanding GPS/GNSS: Principles and Applications.

[28] Evenden G.I., Rouault E., Warmerdam F., Evers K., Knudsen T., Butler H., Taves M.W., Schwehr K., Sales de Andrade E., Karney C. (2024). PROJ(9.4.1) Coordinate Transformation Software Library.

[29] Foote T., Purvis M. (2010). REP 103—Standard Units of Measure and Coordinate Conventions. https://www.ros.org/reps/rep-0103.html.

[30] Salvador S., Chan P. (2007). Toward accurate dynamic time warping in linear time and space. Intell. Data Anal..

[31] Maschinenfabrik Bernard Krone GmbH & Co. KG., LEMKEN GmbH & Co. KG. COMBINED POWERS—The Next Level in Automated Agriculture. https://combined-powers.com/en/.

